# Process Modeling and Micromolding Optimization of HA- and TiO_2_-Reinforced PLA/PCL Composites for Cannulated Bone Screws via AI Techniques

**DOI:** 10.3390/ma18174192

**Published:** 2025-09-06

**Authors:** Min-Wen Wang, Jui-Chia Liu, Ming-Lu Sung

**Affiliations:** 1 Mechanical Engineering Department, National Kaohsiung University of Science and Technology, Kaohsiung 807618, Taiwan; s12025214@gmail.com; 2 Physical Education Office, National Kaohsiung University of Science and Technology, Kaohsiung 811532, Taiwan; smlgreen@nkust.edu.tw

**Keywords:** micromolding, PLA/PCL composites, hydroxyapatite, titanium dioxide, shrinkage behavior, Taguchi method, AI-assisted modeling

## Abstract

A bioresorbable cannulated bone screw was developed using PLA/PCL-based composites reinforced with hydroxyapatite (HA) and titanium dioxide (TiO_2_), two additives previously reported to enhance mechanical compliance, biocompatibility, and molding feasibility in biodegradable polymer systems. The design incorporated a crest-trimmed thread and a strategically positioned gate in the thin-wall zone opposite the hexagonal socket to preserve torque-transmitting geometry during micromolding. To investigate shrinkage behavior, a Taguchi orthogonal array was employed to systematically vary micromolding parameters, generating a structured dataset for training a back-propagation neural network (BPNN). Analysis of variance (ANOVA) identified melt temperature as the most influential factor affecting shrinkage quality, defined by a combination of shrinkage rate and dimensional variation. A hybrid AI framework integrating the BPNN with genetic algorithms and particle swarm optimization (GA–PSO) was applied to predict the optimal shrinkage conditions. This is the first use of BPNN–GA–PSO for cannulated bone screw molding, with the shrinkage rate as a targeted output. The AI-predicted solution, interpolated within the Taguchi design space, achieved improved shrinkage quality over all nine experimental groups. Beyond the specific PLA/PCL-based systems studied, the modeling framework—which combines geometry-specific gate design and normalized shrinkage prediction—offers broader applicability to other bioresorbable polymers and hollow implant geometries requiring high-dimensional fidelity. This study integrates composite formulation, geometric design, and data-driven modeling to advance the precision micromolding of biodegradable orthopedic devices.

## 1. Introduction

Orthopedic implants play a crucial role in modern fracture management and reconstructive surgery. As noted by Middleton and Tipton [1], traditional implants are predominantly made of metals such as stainless steel, titanium alloys, and cobalt–chromium alloys, due to their excellent mechanical strength, corrosion resistance, and biocompatibility. Nevertheless, such metals exhibit an inherent mismatch in Young’s modulus compared to native bone, often leading to stress shielding, periprosthetic bone resorption, and an increased risk of secondary fractures due to mechanical unloading [2]. Additionally, prolonged in vivo exposure can provoke ion release via corrosion and wear, triggering adverse local tissue responses, including inflammation and hypersensitivity [3]. Critically, their non-resorbable nature frequently necessitates secondary surgical removal, contributing to elevated healthcare costs, postoperative morbidity, and patient discomfort [1]. Biodegradable polymers have emerged as viable alternatives, particularly in orthopedic applications that benefit from gradual material resorption. Since the clinical introduction of poly(lactic-co-glycolic acid) sutures in the 1960s [1], a diverse range of bioabsorbable materials—including polylactic acid (PLA), polyglycolic acid (PGA), polycaprolactone (PCL), and polydioxanone (PDS)—have been extensively investigated [1,4]. These polymers permit progressive load transfer to regenerating bone tissue while reducing stress shielding and eliminating the need for implant removal [1]. Despite their promise, monolithic biodegradable polymers often fail to satisfy the multifaceted mechanical and degradation requirements of orthopedic devices. PLA, for instance, exhibits commendable strength and stiffness but suffers from brittleness and slow degradation; in contrast, PCL offers ductility and processability but possesses low strength and similarly slow resorption rates [4].

Material strategies such as polymer blending—e.g., PLA/PCL combinations—have been employed to enhance toughness and modulate degradation behavior, as demonstrated in nanocomposite systems [5]. Additionally, the incorporation of ceramic reinforcements—most notably hydroxyapatite (HA) and titanium dioxide (TiO_2_)—has been shown to enhance mechanical integrity, bioactivity, and degradation profiles in biodegradable composites [6,7]. TiO_2_, in particular, has been reported to improve dispersion, crystallinity, and rheological behavior within PLA/PCL matrices, thereby enhancing processability and dimensional stability [8,9]. Kaseem et al. reviewed further demonstrated that surface-treated TiO_2_ promotes uniform particle distribution and contributes to improved mechanical performance [10].

Against this material backdrop, the present study focuses on the development and process optimization of biodegradable cannulated bone screws—devices featuring internal hollow geometries that facilitate guidewire-assisted placement, a critical requirement in minimally invasive orthopedic procedures [11]. Such screws are commonly employed in ligament reconstruction surgeries, including anterior cruciate ligament (ACL) repair, where they serve to secure grafts within bone tunnels and provide initial mechanical stability during early tissue integration [11]. While interference screws represent a specific subclass of cannulated bone screws, the present work addresses broader design and manufacturing challenges associated with polymer-based cannulated implants [11].

However, the intricate geometry of these devices—including their fine threads, central lumens, and socket interfaces—combined with their strict dimensional tolerances, presents substantial challenges in polymer-based fabrication [12,13,14]. Furthermore, biodegradable polymers such as PLA exhibit high sensitivity to melt temperature, shear rate, and cooling dynamics during processing, making micro-scale molding particularly complex and error-prone [15]. Despite these difficulties, injection molding remains the prevailing manufacturing technique due to its scalability, production efficiency, and near-net-shape capabilities [12,13,14,15].

Nonetheless, standard molding strategies struggle to achieve the geometric precision and flow control required for cannulated screw production. Micro-injection molding offers enhanced resolution, cavity fill precision, and dimensional repeatability, positioning itself as a viable solution [12].

Currently, comprehensive data relating to optimized injection molding parameters for biodegradable cannulated composite screws remain limited. Therefore, exploring correlations between processing conditions and resulting product quality—such as shrinkage and dimensional stability—holds critical value for both clinical performance and manufacturing reliability.

While the present study focuses on PLA/PCL-based systems, the modeling framework—particularly the integration of geometry-specific gate design and normalized shrinkage prediction—offers broader applicability to other bioresorbable polymers and implant geometries. This generalizability is especially relevant for hollow or threaded components requiring high-dimensional fidelity, where conventional process control often falls short.

Building on previous integrated optimization frameworks [16,17], in this study, a hybrid modeling approach is developed to enhance injection molding process control. A Taguchi L9 orthogonal array is first employed—not for direct process optimization—but to generate a uniformly distributed training dataset that captures the relationship between molding parameters and cannulated screw quality indicators [18]. This structured experimental design facilitates robust training of a back-propagation neural network (BPNN), which is subsequently validated as a predictive model for shrinkage behavior. To further refine process control, genetic algorithm (GA) and particle swarm optimization (PSO) frameworks are integrated to identify global optima for targeted performance characteristics [17,19].

In summary, this work aims to optimize the injection molding process for PLA/PCL-based biodegradable cannulated bone screws, incorporating either hydroxyapatite (HA) or titanium dioxide (TiO_2_) ceramic fillers to enhance biocompatibility and structural performance [6,7,8,10]. Through this hybrid modeling approach—combining Taguchi-based data generation, BPNN prediction, and GA–PSO metaheuristic optimization—this research establishes a comprehensive methodology not only for high-performance, dimensionally stable implant production, but also for transferable process strategies applicable to broader classes of bioresorbable devices.

## 2. Experimental Methods

### 2.1. Biodegradable Cannulated Bone Screw Design

**Figure 1:**
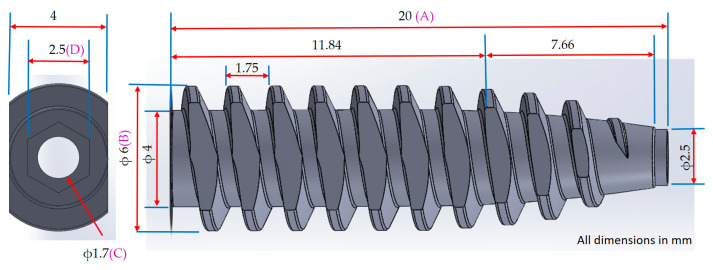
Dimensional design and shrinkage measurement points of a cannulated bone screw. The figure illustrates the key dimensional parameters of the cannulated bone screw, including total length (A), thread outer diameter (B), bore diameter (C), and hex socket width (D). These labeled positions also serve as reference points for post-molding shrinkage measurements.

A novel cannulated bone screw, illustrated in Figure 1, was designed for biodegradable orthopedic applications using PLA/PCL-based composites. The screw measured 20 mm in overall length, featuring an outer thread diameter of 6.0 mm and a central bore of 1.7 mm. The proximal segment—defined herein as the internal hexagonal socket region—extended over 11.84 mm with a consistent thread root diameter of 4.0 mm, yielding a maximum wall thickness of 1.15 mm. A tapered segment spanned the remaining 7.66 mm, beginning after a short 0.5 mm transition zone with a constant outer diameter of 2.5 mm. The taper, defined by a 3.25° angle, reduced the thread root diameter linearly to 2.55 mm and resulted in a minimum wall thickness of 0.4 mm.

The thread profile adopted an R-type geometry, featuring rounded crests and roots with a pitch of 1.75 mm. Rounded thread profiles are commonly employed in polymeric orthopedic screws to reduce stress concentrations and improve moldability, as smoother transitions between crests and roots help minimize localized strain during insertion and enhance flow behavior during injection molding [20]. Crest trimming was applied continuously along both lateral flanks of the threaded segment, extending from the proximal to distal ends and reaching down to the thread root. This full-length bilateral flattening formed planar surfaces that facilitate mold separation in a three-plate injection setup, eliminating the need for rotating cores. This geometric adaptation supports mold release and has been shown to preserve fixation strength when core and flank geometry are maintained. Yu et al. [21] demonstrated that thread depth critically influences insertion torque and pull-out strength. Although crest trimming was not investigated directly, their results suggest that preserving thread depth and flank integrity may limit mechanical compromise. Feng et al. [22] further confirmed that thread profile geometry and depth significantly govern axial pull-out and lateral migration resistance, underscoring the importance of deliberate profile design to ensure biomechanical performance.

An internal hexagonal drive socket (2.5 mm across flats, 2.0 mm depth) was integrated into the screw head to enable guidewire-based surgical insertion. The distal taper and crest-trimmed thread profile were implemented to accommodate mold release and surgical compatibility without compromising mechanical integrity. Gating and mold separation strategies were designed to preserve torque-bearing features near the proximal socket. The technical details are provided in Section 2.4.

### 2.2. Preparing Material

This study employed PLA/PCL composites reinforced with either hydroxyapatite (HA) or titanium dioxide (TiO_2_) to fabricate the bone screw depicted in Figure 1. Compared to pure PLA, PLA/PCL blends exhibit enhanced elongation and impact toughness, albeit with slightly reduced tensile strength. The prior literature indicates that PLA/PCL at an 80/20 weight ratio achieves optimal balance in ductility and toughness [23]. Therefore, this blend was selected as the base matrix for all formulations in this study, using PLA (Luminy^®^ L175, Corbion Purac Group, Gorinchem, The Netherlands) and PCL (Capa 6800, Perstorp, Perstorp, Sweden).

Ceramic fillers were incorporated to further enhance the mechanical and biological performance of the composite. HA (Sigma-Aldrich, distributed by Heng Chang Yuan Materials Co., Taiwan, China) has been widely shown to improve strength and stiffness in biodegradable polymer matrices, particularly in orthopedic applications [24,25]. TiO_2_ nanoparticles (Titanium (IV) oxide, Aeroxide^®^ P25, supplied by Jing Ming Chemical Co., Taiwan, China), on the other hand, have demonstrated beneficial effects on thermal stability, dispersion uniformity, crystallization behavior, and tensile strength in PLA- and PCL-based systems [9,26,27]. Accordingly, two sets of composite formulations were developed:

- PLA/PCL (80/20 wt%) + 10, 20, 30 wt% HA.
- PLA/PCL (80/20 wt%) + 1, 3, 5 wt% TiO_2_.

**Figure 2:**
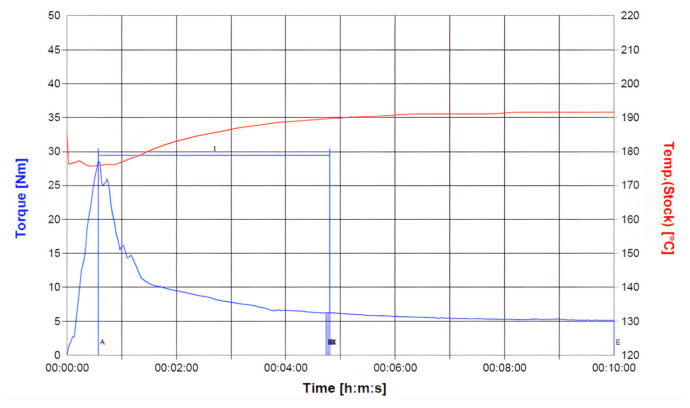
Torque and temperature curve of PLA/PCL (80%:20%) with 10 wt% HA during mixing process.

**Figure 3:**
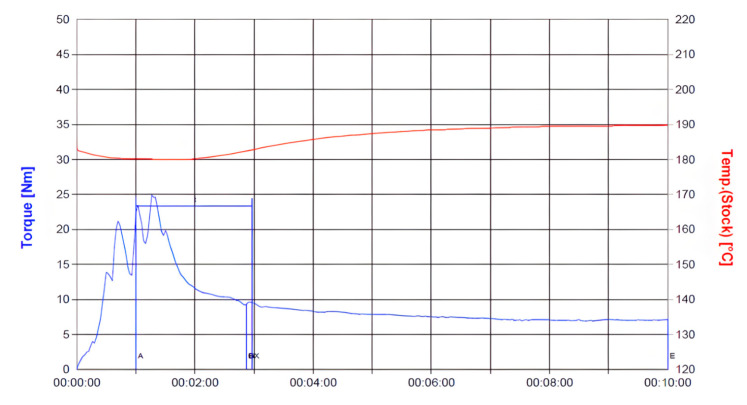
Torque and temperature curve of PLA/PCL (80%:20%) with 3 wt% TiO_2_ during mixing process.

Material compounding was conducted using a Brabender (Plasti-Corder Lab-Station, Duisburg, Germany) at 190 °C and 50 rpm for 10 min to ensure homogeneous dispersion. The temperature and torque profiles, illustrated in Figure 2 and Figure 3, confirm steady-state processing, facilitating consistent filler distribution within the PLA/PCL matrix. During the initial mixing phase, a transient drop in barrel temperature was observed due to the significant heat uptake required to initiate polymer and filler melting. This was followed by torque stabilization, indicating the attainment of uniform mixing conditions. Through this material preparation methodology, this study aimed to evaluate how varying HA and TiO_2_ contents affect the mechanical properties and processing behavior of PLA/PCL composites—laying the foundation for the subsequent optimization and application of biodegradable cannulated bone screws.

Although direct microstructural analysis of filler dispersion was not conducted in this study, the stabilized torque and temperature profiles suggest homogeneous mixing. We acknowledge that torque-based inference alone may be insufficient to confirm nanoparticle dispersion, particularly for TiO_2_ and HA fillers. Future work will incorporate quantitative dispersion metrics, including microscopy and image-based analysis, to validate ceramic filler distribution in PLA/PCL matrices.

### 2.3. Tensile Test Sample Molding

To evaluate the mechanical performance of PLA/PCL composites containing varying concentrations of HA and TiO_2_, tensile specimens were fabricated and tested in accordance with ASTM D638 Type V [28] standards. Each specimen measured 63.5 mm in length, 9.53 mm in width, and 3.2 mm in thickness [28]. After compounding via the Brabender Plasti-Corder (as described previously), the materials were pelletized and subsequently molded into test specimens using a Ling Fong 30 Ton compression press (Tainan, Taiwan, China). Compression molding was conducted at 200 °C under 150 kg/cm^2^ for 3 min, followed by cooling to 60 °C using a circulating water system to ensure dimensional stability.

**Figure 4:**
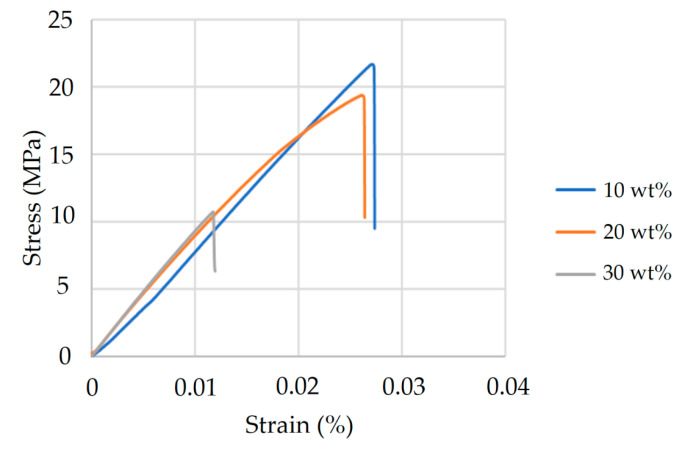
Tensile strength and elongation of PLA/PCL composites with varying HA content. Specimens containing 10 wt%, 20 wt%, and 30 wt% HA were tested according to ASTM D638 Type V standards. The 10 wt% HA formulation exhibited the highest tensile strength and elongation, suggesting optimal particle dispersion and effective reinforcement. A marked decline in mechanical properties was observed at 30 wt% HA, which may be attributed to reduced matrix continuity and filler overloading.

**Figure 5:**
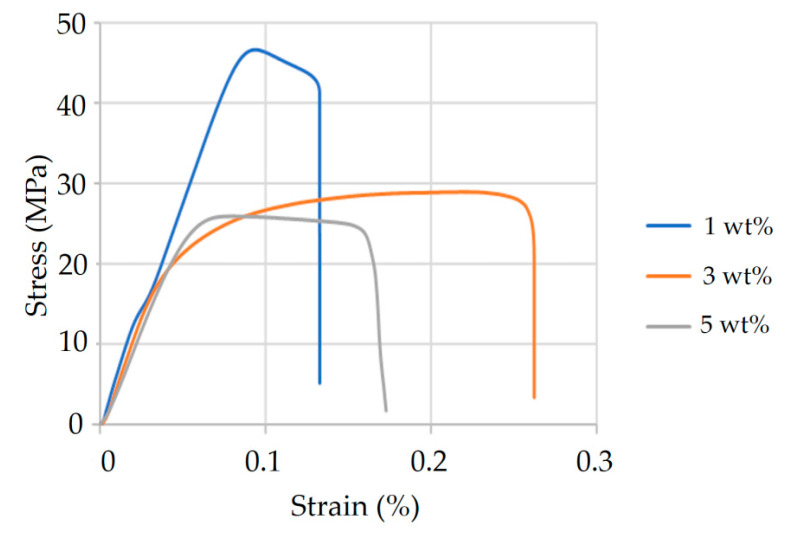
Tensile strength and elongation of PLA/PCL composites with varying TiO_2_ content. Samples containing 1 wt%, 3 wt%, and 5 wt% TiO_2_ were evaluated. The 1 wt% group exhibited the highest tensile strength but limited ductility. In contrast, the 3 wt% formulation achieved improved elongation with moderate strength, representing the most favorable balance of toughness and resilience. Both strength and ductility declined notably in the 5 wt% group, possibly due to filler overloading and reduced interfacial compatibility.

Mechanical characterization was performed using a YM-H51 Universal Testing Machine (Yang Yi Technology Co., Tainan, Taiwan, China). Four tensile specimens were tested for each composition. Due to the exploratory nature of the original study, only one representative stress–strain curve per formulation was retained and presented, being selected based on its consistency with the overall mechanical behavior observed across samples. The corresponding tensile strength and elongation data for HA- and TiO_2_-modified formulations are also shown in Figure 4 and Figure 5. The HA-enhanced composites achieved optimal performance at 10 wt% HA, demonstrating the highest tensile strength and elongation; this composition was therefore selected for subsequent injection molding trials. In contrast, TiO_2_-modified composites exhibited their highest peak stress at 1 wt%, but their limited strain-to-failure rate indicated poor ductility. The 3 wt% TiO_2_ formulation achieved a more favorable balance—exhibiting moderate tensile strength with significantly improved elongation—whereas the 5 wt% sample showed a decline in both strength and ductility. As a result, 3 wt% TiO_2_ was selected as the preferred filler concentration, prioritizing toughness over peak strength for orthopedic applications.

The selection of PLA/PCL at an 80/20 ratio was guided by the prior literature [23], which reported an optimal elastic modulus within this range. Studies have shown that increasing PCL content enhances ductility while reducing strength, making the 80/20 composition a suitable structural base for filler modifications. These formulation decisions ensure both biomechanical viability and processability for micro-scale injection molding trials.

### 2.4. Injection Molding Experiment

Injection molding was employed to fabricate the cannulated bone screws using PLA/PCL-based biodegradable composites, utilizing a Battenfeld Microsystem 50 micromolding machine (Wittmann Battenfeld GmbH, Kottingbrunn, Austria). Given the part’s micro-scale geometry and thin-wall features, a custom-designed three-plate mold system was implemented to ensure dimensional fidelity and facilitate demolding. The mold incorporated a pin gate located at the runner inlet, initiating melt flow in a three-plate configuration. Downstream, the melt was directed through a disk-shaped diaphragm gate positioned at the distal thin-wall region, enabling uniform flow distribution into the screw cavity. During mold opening, the stripper plate facilitated automatic separation of the pin gate and runner system from the molded part. The diaphragm gate, which remained attached to the distal thin-wall region, was manually removed post-molding to preserve cavity integrity and minimize shear-induced residuals.

While injection molding enables high-throughput replication, the dimensional accuracy and mechanical reliability of molded components are strongly governed by processing conditions. To generate a representative and evenly distributed training dataset for AI modeling, in this study, we employed a Taguchi L9 orthogonal array not as a direct optimization tool, but as a structured experimental strategy to vary the molding parameters across a balanced design space. Melt temperature, injection speed, and holding speed were selected as the primary control factors based on the prior literature [29,30], which highlighted their influence on viscosity, cavity filling, molecular orientation, and cooling shrinkage.

Shrinkage rate was defined as the main quality metric. A total of 14 experimental trials were performed: 9 based on the Taguchi L9 design, and 5 additional runs within the process window to expand the predictive dataset. The L9 data were used to train a back-propagation neural network (BPNN), which served as a predictive model for shrinkage rate. Five validation trials were subsequently conducted to assess prediction fidelity.

Once trained, the BPNN was integrated into a hybrid optimization framework using a genetic algorithm (GA) and particle swarm optimization (PSO). These metaheuristic techniques iteratively refined the model, converging on optimal molding parameters that minimize shrinkage. Importantly, the optimization process did not rely on Taguchi-derived parameter combinations, but rather leveraged AI-assisted prediction and refinement to achieve superior shrinkage control and dimensional stability. This approach demonstrated the enhanced process robustness and mechanical reliability of the final molded cannulated bone screws.

#### Experimental Parameter Ranges for AI Training via Taguchi Design

Prior to implementing the Taguchi experiment, a series of trial moldings was conducted to empirically define the processing windows for key molding parameters. To ensure reproducibility and stable part formation, each parameter was incrementally adjusted from conservative baseline values, and molding outcomes were evaluated based on defect occurrence and repeatability. Since both HA- and TiO_2_-enhanced PLA/PCL composite formulations exhibited similar flow behavior and thermal response profiles, a unified parameter range was adopted for orthogonal experimentation.

For melt temperature, trial moldings were performed in 5 °C increments starting from 180 °C. Temperatures below 190 °C led to premature solidification and short shots, whereas temperatures exceeding 210 °C promoted sink mark deformation. Stable molding was defined by consistent part formation without critical defects, and the acceptable temperature range was defined as 190–210 °C.

**Figure 6:**
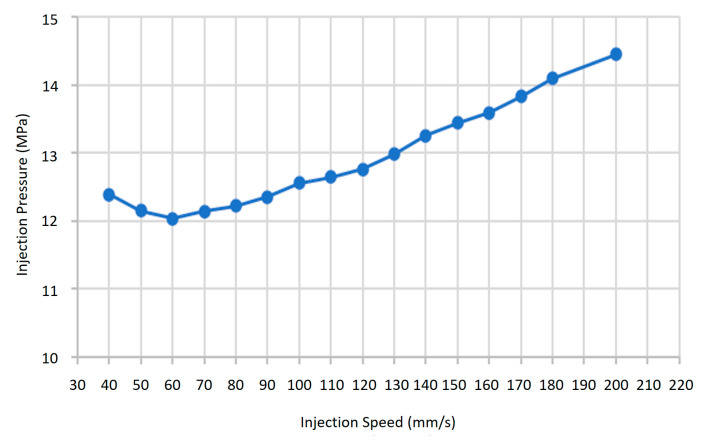
Injection speed–pressure profile simulated via Moldex3D for PLA/PCL blend without additives. Results support the selection of a 40–80 mm/s range for stable injection molding.

Regarding injection speed, moldings were conducted in 5 mm/s steps starting from 30 mm/s. Speeds below 40 mm/s caused incomplete mold filling, while speeds above 200 mm/s induced flash formation. To further support parameter selection and define fixed conditions for Taguchi experimentation, a mold flow simulation using a neat PLA/PCL blend (excluding HA or TiO_2_) was conducted via Moldex3D (CoreTech System Co., Ltd., Zhubei City, Hsinchu County, Taiwan, China). The simulation served three purposes: (1) assisting in the preliminary selection of the injection speed range based on pressure trends, (2) estimating packing time via gate solidification analysis, and (3) evaluating cooling time based on cavity temperature uniformity (ΔT < 10 °C). The simulation incorporated standard boundary conditions commonly used in injection molding analysis, including fixed mold wall temperatures, uniform melt inflow at the sprue entrance, and no-slip conditions at cavity surfaces. The thermal and rheological properties of the neat PLA/PCL blend were experimentally measured and manually input into Moldex3D to ensure accurate material representation. These settings enabled realistic flow behavior and pressure predictions for parameter screening. The resulting injection speed–pressure profile is shown in Figure 6. As the injection speed increased from 40 mm/s to 60 mm/s, the required pressure slightly decreased. However, beyond 80 mm/s, the pressure increased significantly with speed, and continued rising until reaching the simulated limit of 200 mm/s. These trends suggest that excessively high injection speeds demand greater filling pressure and may cause flash, while moderate speeds (40–80 mm/s) enable more efficient and stable flow. Thus, considering both the trial moldings and simulation findings, the injection speed range was conservatively defined as 40–80 mm/s, while packing and cooling times were fixed based on the simulation outputs to ensure consistency across Taguchi trials.

For the selection of holding speed, moldings were performed in 3 mm/s increments starting from 4 mm/s. Values lower than 10 mm/s failed to sustain adequate cavity packing, leading to insufficient compensation during cooling. Stable molding was observed from 10 mm/s up to the machine’s maximum setting of 33 mm/s. However, to avoid operating near the upper hardware limit, the acceptable range was conservatively defined as 10–30 mm/s.

Exactly 10 moldings were performed for each parameter combination to confirm repeatability and observe defect trends. These experimentally validated process windows were discretized into three levels for each control factor—melt temperature, injection speed, and holding speed—and used to construct a Taguchi L9 orthogonal array. This array was not intended for direct process optimization, but rather served as a structured method to generate a balanced and representative dataset for AI model training, as detailed in Table 1.

**Figure 7:**
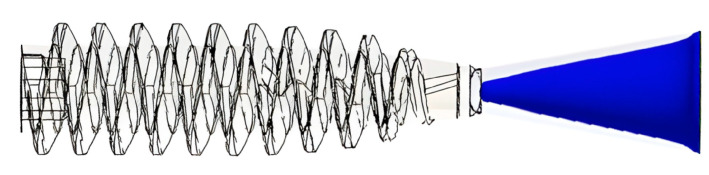
Moldex3D simulation showing gate solidification at 2.635 s during the packing phase. Based on this result, the holding time was conservatively set to 3 s to ensure complete packing and dimensional stability.

**Figure 8:**
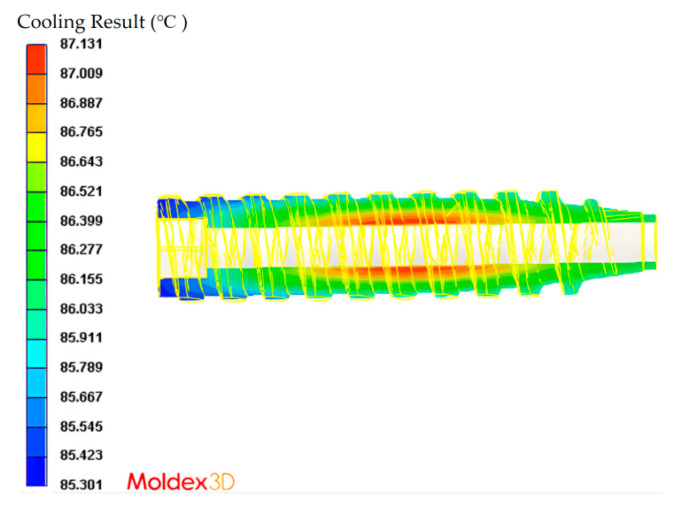
Temperature distribution within the polymer melt inside the cannulated bone screw mold after 10 s of cooling, as simulated using Moldex3D 2020. The maximum and minimum temperatures were 87.131 °C and 85.301 °C, respectively, yielding a cavity temperature difference of 1.83 °C. These results satisfy the cooling uniformity criterion (ΔT < 10 °C) and support the selection of 10 s as the baseline cooling time.

Fixed molding parameters were defined to ensure consistency across experimental trials (Table 2). Moldex3D mold flow simulations indicated gate solidification at 2.635 s (Figure 7), and the holding time was conservatively set to 3 s to ensure complete packing. Cooling time was initially set to 10 s based on thermal simulation results (Figure 8), which showed the melt temperature difference in the cavity of only 1.83 °C—well below the 10 °C uniformity threshold. To accommodate variable melt temperatures during Taguchi trials, the cooling time was extended to 15 s for thermal stabilization. Back pressure was fixed at 40 bar, as lower values were found to induce voids in molded parts, likely due to the occurrence of trapped air or moisture during melt preparation.

Importantly, maintaining cavity temperature gradients below 10 °C helps prevent post-ejection thermal reflow, which can lead to defects such as warpage and sink marks. This design strategy aligns with those of prior studies on cooling uniformity and dimensional control in injection molding processes [31,32].

Based on the defined factor levels, a Taguchi L9 orthogonal array was constructed (Table 3). Injection molding was performed for each parameter combination following a 30 min stabilization period. Melt and mold temperatures were monitored continuously, maintaining deviations within ±1 °C.

Following the molding process, three cannulated bone screw specimens were randomly selected from each trial and conditioned at room temperature for three days prior to measurement. Dimensional evaluations included overall length (A), thread outer diameter (B), bore diameter (C), and hex socket width (D), as illustrated in Figure 1.

Shrinkage rates in the axial (length), radial (outer diameter), and internal (bore and socket width) directions were calculated using the following equation:

Shrinkage Rate (%) = (Mold Size − Product Size)/Mold Size × 100%(1)

The standard deviation, *S*, of the measured shrinkage rates was calculated using the following sample-based formula:

(2) $$S=\sqrt{\frac{\sum_{i=1}^{n}{\left(X_{i}\;-\;\bar{X}\right)}^{2}}{n\;-\;1}}.$$

where *X_i_* is the shrinkage rate of the *i^th^* measurement, $\bar{X}$ is the mean shrinkage, and n is the number of measurements per trial (n = 3).

### 2.5. Experiment for Neural Network Optimization

To refine the injection molding parameters for biodegradable PLA/PCL-based cannulated bone screws, this study involved an artificial intelligence (AI)-driven optimization framework focused on shrinkage rate control. Dimensional shrinkage was selected as the primary quality metric, enabling streamlined predictor construction and an optimization search.

The strategy consisted of two stages: (1) developing a molding quality predictor using a back-propagation neural network (BPNN) and (2) implementing a hybrid metaheuristic search combining a genetic algorithm (GA) and particle swarm optimization (PSO) to identify process parameters that minimize shrinkage.

The Taguchi L9 experimental dataset was used to train the neural network, and five additional molding trials—based on random parameter combinations within the original control factor range—served as test data to validate predictive accuracy. Several process parameters, including melt temperature, injection speed, and holding speed, were adopted as input variables, while the normalized shrinkage rate served as the sole output response. This enabled the development of a quality predictor for shrinkage performance.

The core architecture of the neural network and the hybrid optimization framework was adapted from Chen and Kurniawan [17], whose methodology remains technically robust and widely applicable in molding process studies. In the present study, targeted modifications were introduced to address the specific objective of minimizing shrinkage in fabricating biodegradable cannulated bone screws. These modifications included the simplification of quality input parameters, a single-output predictor design, and a redefined fitness function focused solely on dimensional accuracy. While the foundational structure aligns with prior work [17], the model described herein features domain-specific adaptations that enable precise shrinkage control for medical-grade biodegradable components.

#### 2.5.1. BPNN Molding Quality Predictor

Following the Taguchi L9 experiments, all the input factors—melt temperature, injection speed, and holding speed—along with the output quality indicator (shrinkage rate) were normalized to enhance model convergence and prevent saturation near the boundaries of the sigmoid activation function. A linear scaling approach was adopted, bounding all normalized values within the range of 0.1 to 0.9.

Normalization was performed for the input control parameters in a direct manner: lower values were mapped to 0.1 and higher values to 0.9, preserving their natural order. In contrast, the shrinkage rate, being a “smaller-is-better” target, was normalized in reverse—such that lower shrinkage values (i.e., more desirable values) were mapped to 0.9 and higher values to 0.1. This directional adjustment ensured that higher normalized values consistently represented more desirable outcomes across both input and output domains, thereby aligning model interpretation with the optimization objective.

Notably, only the normalized shrinkage rate was selected as an output feature for model training. Variance-based metrics, such as standard deviation and signal-to-noise ratio (S/N), were excluded to maintain predictor simplicity and ensure consistency in the learning process.

A three-layer feedforward neural network was constructed with the following structure:

- Input layer: 3 neurons (control parameters).
- Hidden layer: 8 neurons trained using the scaled conjugate gradient method.
- Output layer: 1 neuron (normalized shrinkage rate), with sigmoid activation.

**Figure 9:**
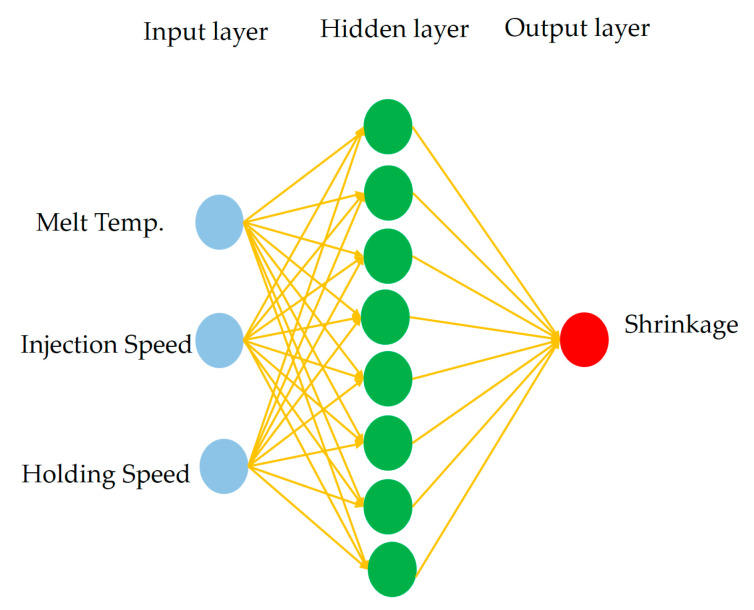
Architecture of the BPNN shrinkage predictor. The network consists of three input neurons corresponding to melt temperature, injection speed, and holding speed; a hidden layer with eight neurons; and one output neuron representing the normalized shrinkage rate.

The training set consisted of nine trials from the Taguchi orthogonal array, while five additional molding runs—based on random combinations within the process window—served as the test data. These test runs were not part of the original array but rather designed to validate the neural network’s ability to generalize shrinkage prediction across feasible molding conditions. All neural network training, prediction, and visualization procedures—including regression analysis and error convergence plots—were performed using MATLAB R2020a (MathWorks Inc., Natick, MA, USA). The BPNN model and optimization framework were implemented via custom scripts adapted from Chen and Kurniawan [17], without relying on built-in toolboxes. The finalized architecture is illustrated in Figure 9.

#### 2.5.2. Optimization Framework Using GA–PSO

**Figure 10:**
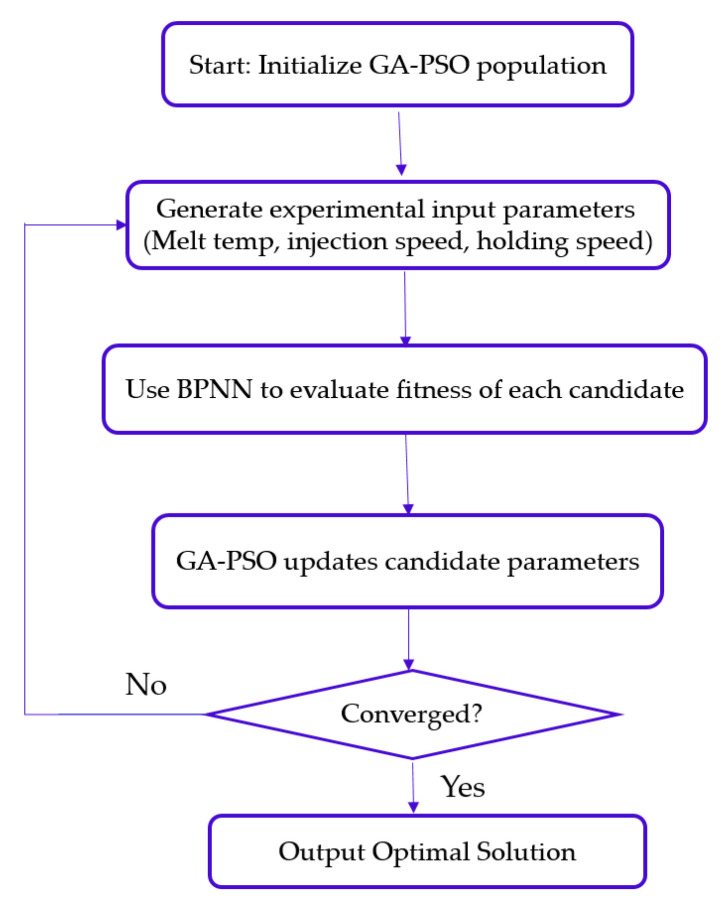
Conceptual overview of the GA–PSO hybrid optimization process, which was adapted from existing variants to suit the specific molding requirements in this study. The flowchart highlights the iterative interaction between BPNN prediction and parameter refinement, enabling an efficient search for optimal shrinkage performance without relying on gradient-based methods.

The trained BPNN model was embedded within a hybrid optimization framework that combined a genetic algorithm (GA) and particle swarm optimization (PSO). This integration enabled efficient exploration and refinement across the multidimensional design space of molding parameters. The overall optimization procedure is illustrated in Figure 10, which outlines the interaction between BPNN-based prediction and metaheuristic search strategies.

Since the BPNN predictor was specifically designed to estimate the normalized shrinkage rate, the optimization objective was aligned accordingly. The fitness function was formulated to minimize the deviation between the predicted normalized shrinkage value and a reference target of 0.1 (based on normalized data). The objective function is expressed as

min *F*(*Y*) = (*Q*(*Y*) − 0.1)^2^ (3)

where

*Y* = {*y*_1_, *y*_2_, *y*_3_}: process parameter vector;

*y*_1_: melt temperature (190–210 °C);

*y*_2_: injection speed (40–80 mm/s);

*y*_3_: holding speed (10–30 mm/s);

*Q*(*Y*): normalized shrinkage rate predicted by the BPNN;

*F*(*Y*): fitness function value representing the squared deviation from the target shrinkage rate.

This squared-error formulation enabled gradient-free optimization while maintaining consistency with the output range of the neural network. Through iterative refinement, the GA–PSO hybrid search progressively adjusted the process settings to approach the lowest achievable shrinkage under realistic molding conditions.

## 3. Results and Discussion

### 3.1. Thermal and Rheological Properties of PLA/PCL Matrix

**Figure 11:**
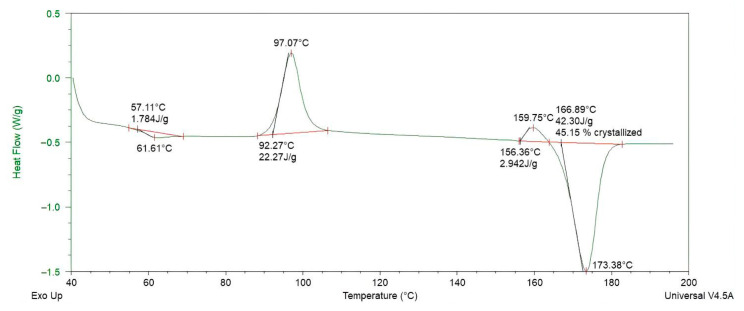
Differential scanning calorimetry (DSC) thermogram of PLA/PCL (80:20 wt%) blend, showing the melting transition of PLA at 173.38 °C and the thermal behavior of the composite.

To establish a reliable baseline for subsequent composite comparisons, the thermal and rheological characteristics of the PLA/PCL (80:20 wt%) blend were first examined. Differential scanning calorimetry (TA Instruments DSC-Q10, New Castle, DE, USA) revealed two distinct melting transitions: one near 61.61 °C corresponding to PCL, and another at 173.38 °C attributed to PLA, indicating that complete melting of both components requires temperatures exceeding 173.38 °C (Figure 11).

**Figure 12:**
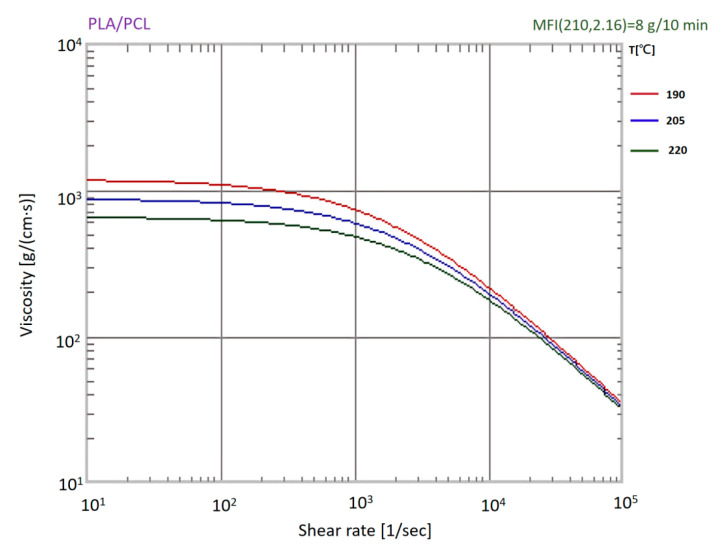
Shear viscosity profiles of PLA/PCL (80:20 wt%) blend at 190 °C, 205 °C, and 220 °C, showing temperature-dependent shear-thinning behavior.

Rheological measurements were performed using a capillary rheometer (RHEO-TESTER 1000, Göttfert Werkstoff-Prüfmaschinen GmbH, Buchen, Germany) at 190 °C, 205 °C, and 220 °C—temperatures selected to reflect typical plasticization conditions for PLA-based materials. The resulting shear viscosity profiles (Figure 12) exhibit temperature-dependent shear-thinning behavior. Since the PLA/PCL blend is not included in the Moldex3D default material library, a custom rheological model was constructed from the experimental data to support mold flow simulation and processing window optimization.

### 3.2. Taguchi-Based Evaluation of Molding Parameters

To evaluate the shrinkage behavior of PLA/PCL composites incorporating ceramic fillers, an L9 (3^3^) Taguchi orthogonal array experiment was conducted for two formulations: PLA/PCL/HA and PLA/PCL/TiO_2_. The control factors investigated were melt temperature, injection speed, and holding speed. Cannulated bone screws were molded under these combinations, and post-molding dimensions were measured using a laser scanning microscope (VK-X1100 model, KEYENCE Corporation, Osaka, Japan) to quantify linear shrinkage and dimensional variation.

A composite quality index—defined as the sum of the shrinkage rate and standard variation—was employed. This index reflects both average shrinkage and its variability, aligning with the dimensional precision required for biomedical applications. This formulation prioritizes dimensional consistency, making it suitable for a “smaller-the-better” Taguchi analysis. Signal-to-noise (S/N) ratio transformations were applied to facilitate parametric comparison and assess the sensitivity of molding quality to processing conditions. Response tables and variance analyses were used to identify dominant factors influencing shrinkage.

#### 3.2.1. PLA/PCL/HA Composite

**Figure 13:**
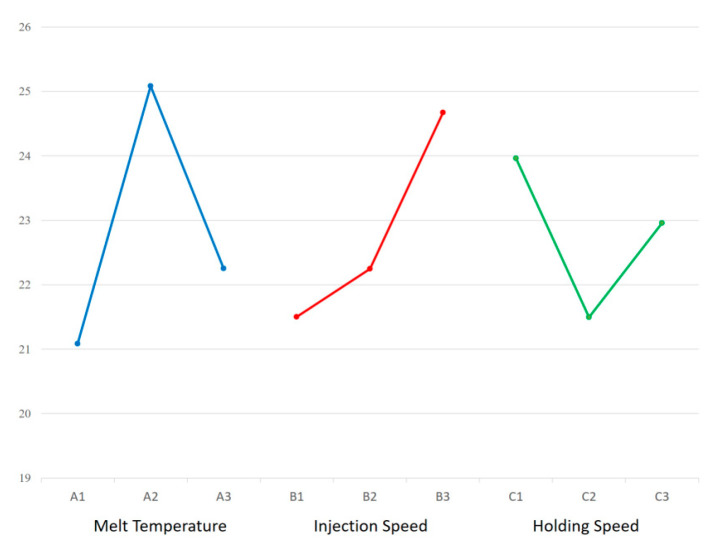
Factor response plots for PLA/PCL/HA system. The optimal parameter combination (A2B3C1) was identified based on the highest S/N ratio.

Measurement data, shrinkage rates, standard deviations, shrinkage–variation sums, and S/N ratios for the HA system are summarized in Table 4 and Table 5. Based on the factor response plots (Figure 13), the parameter combination yielding the best shrinkage performance was identified as A2B3C1, which corresponded to the following values:

- Melt temperature: 200 °C.
- Injection speed: 80 mm/s.
- Holding speed: 10 mm/s.

**Table 4:** Dimensional measurements of PLA/PCL/HA bone screws (unit: mm).

Trial	Specimen	Length	Thread Outer Diameter	Bore Diameter	Hex Socket Width
L1	#1	20	5.923	1.695	2.315
	#2	19.8	5.953	1.696	2.329
	#3	19.834	5.933	1.664	2.436
L2	#1	19.697	5.927	1.687	2.321
	#2	19.741	5.953	1.68	2.4
	#3	19.734	5.932	1.683	2.324
L3	#1	19.887	5.885	1.672	2.38
	#2	19.822	5.922	1.668	2.393
	#3	19.882	5.861	1.671	2.407
L4	#1	19.882	5.976	1.655	2.37
	#2	19.709	5.892	1.653	2.393
	#3	19.864	5.854	1.662	2.419
L5	#1	19.905	5.967	1.682	2.448
	#2	19.904	5.917	1.665	2.461
	#3	19.865	5.939	1.672	2.441
L6	#1	19.82	5.968	1.683	2.489
	#2	19.907	5.917	1.682	2.494
	#3	19.886	5.939	1.672	2.482
L7	#1	19.907	5.926	1.673	2.436
	#2	19.926	5.875	1.68	2.402
	#3	19.944	5.882	1.652	2.385
L8	#1	19.525	5.924	1.664	2.396
	#2	19.709	5.945	1.654	2.458
	#3	19.663	5.915	1.654	2.4
L9	#1	19.963	5.954	1.669	2.45
	#2	19.945	5.983	1.662	2.41
	#3	19.965	5.972	1.675	2.34

**Table 5:** Summary of shrinkage rates, standard deviations, shrinkage–variation sums, and S/N ratios for PLA/PCL/HA composite trials.

Run	Total Shrinkage (%)	Total SD(%)	Shrinkage + SD (%)	S/N
L1	8.267	5.071	13.338	21.653
L2	9.574	2.346	11.92	20.378
L3	8.693	1.659	10.352	21.216
L4	9.348	2.178	11.526	20.585
L5	5.373	1.599	6.972	25.395
L6	3.44	1.039	4.479	29.268
L7	7.704	2.496	10.2	22.265
L8	8.945	2.467	11.412	20.968
L9	6.666	2.759	9.425	23.522

According to the ANOVA results (Table 6), melt temperature emerged as the most influential factor, contributing 37.7% to the observed shrinkage variation, followed by injection speed (24.44%). The lowest shrinkage rate observed, 13.73%, was achieved in trial L6, which coincidentally matched the A2B3C1 condition.

Notably, the error term accounted for 24.13% of the total variance, indicating that a substantial portion of the shrinkage variation was not explained by the selected control factors. This elevated residual error may reflect the presence of unmodeled interactions between processing parameters—such as melt temperature and holding speed—or the influence of latent variables, such as mold temperature or cooling time, that were not included in this design. These findings indicate that the HA system exhibits considerable variability in response to processing conditions, as reflected by the elevated residual error. A comparative assessment with the TiO_2_ composite is presented in the following section.

#### 3.2.2. PLA/PCL/TiO_2_ Composite

The second experimental set focused on TiO_2_-enhanced PLA/PCL composites. Dimensional measurements obtained from molded bone screws are presented in Table 7, while shrinkage rates, standard deviations, shrinkage–variation sums, and S/N ratios are summarized in Table 8.

**Figure 14:**
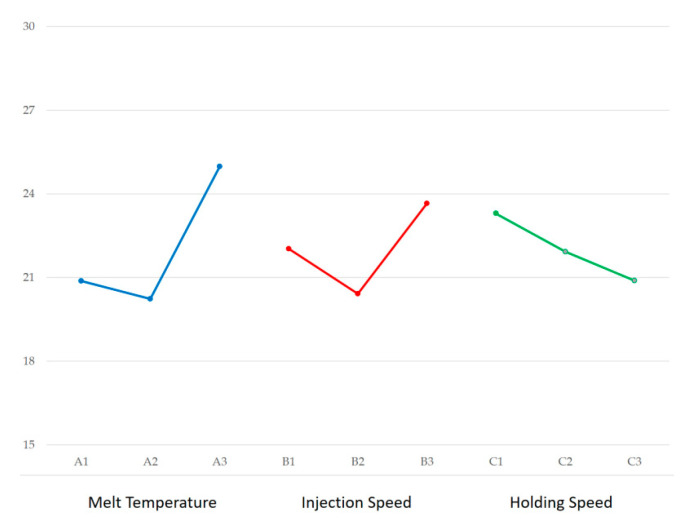
Factor response plots for PLA/PCL/TiO_2_ system. The optimal parameter combination (A3B3C1) was identified based on having the highest S/N ratio.

Based on the factor response plots (Figure 14), the optimal parameter combination was identified as A3B3C1, corresponding to the following values:

- Melt temperature: 210 °C.
- Injection speed: 80 mm/s.
- Holding speed: 10 mm/s.

The Taguchi L9 orthogonal array was employed solely to generate training data for the BPNN model. Among the nine experimental trials conducted, L9 (A3B3C2) yielded the lowest shrinkage rate of 4.25%, serving as a strong reference point. Although the predicted optimal combination based on factor response plots was A3B3C1, this condition was not experimentally tested, as the focus of this study was to pursue further optimization through AI-based methods. The subsequent optimization process using BPNN integrated with GA–PSO is detailed in Section 3.2.

According to the ANOVA results (Table 9), melt temperature was the dominant factor, contributing 58.25% of the total variance to shrinkage variation, followed by injection speed (23.02%) and holding speed (12.74%).

Notably, the error term accounted for only 6.00% of the total variance, indicating that the selected control factors effectively captured the shrinkage behavior of the TiO_2_ composite. This low residual error suggests that the selected control factors effectively captured the dominant sources of shrinkage variation. It also implies limited influence from potential factor interactions or latent variables—such as mold temperature or cooling time—that were not included in this experimental design. Compared to the HA system, the TiO_2_ composite exhibited a more systematic and predictable response to processing conditions.

### 3.3. Results of Quality Predictors

#### 3.3.1. Network Architecture and Data Normalization

Using the framework outlined in Section 2.5, back-propagation neural network (BPNN) models were developed to predict the normalized shrinkage rates of PLA/PCL/HA and PLA/PCL/TiO_2_ composites. Each model incorporated three input variables—melt temperature, injection speed, and holding speed—and a single output neuron representing shrinkage performance. To capture nonlinear interactions among process parameters, a hidden layer comprising eight neurons was implemented.

All training inputs were derived from the L9 Taguchi experimental design and normalized to a range between 0.1 and 0.9 to enhance model stability and convergence. Detailed configuration parameters—including activation functions, training algorithms, and learning rate settings—are summarized in Table 10.

To evaluate the model’s generalization capability, five additional parameter combinations were generated within the original design space and used as validation inputs (Table 11). Complete normalized datasets for both training and validation trials are presented in Table 12 and Table 13, respectively.

It is worth noting that the shrinkage performance index used in the Taguchi analysis—defined as the sum of shrinkage rate and standard deviation—was adopted to reflect both dimensional accuracy and consistency. However, in the BPNN modeling phase, only the normalized shrinkage rate was used as the output variable. While the standard deviation was calculated from only three specimens per trial, limiting statistical robustness, this composite metric remains suitable for guiding parameter selection within the Taguchi framework, whose primary objective is to explore process sensitivity and define robust operating ranges. Preliminary repeatability tests conducted during the early phase of this study confirmed low intra-group variation in shrinkage rate, thereby supporting the use of three specimens per trial for comparative screening within the Taguchi framework. Although this sample size limited statistical power, it was deemed sufficient for identifying process trends. Expanded sampling strategies have since been developed to improve model robustness in subsequent investigations.

Importantly, the BPNN model was not trained using standard deviation values and was therefore not directly influenced by statistically limited variance metrics. This separation between Taguchi-based screening and AI model training ensured that the predictive framework remained focused on normalized shrinkage trends rather than composite variability indices.

The AI-predicted parameter combinations were derived through interpolation within the Taguchi design space, rather than direct replication of existing experimental groups. This approach enabled the identification of shrinkage-optimal settings not explicitly tested in the original L9 matrix, thereby extending the utility of the Taguchi framework through data-driven refinement.

While the validation of AI-optimized parameters was also conducted using three specimens per formulation, this approach was consistent with the comparative screening strategy adopted throughout this study. The observed consistency in shrinkage behavior across trials supports the feasibility of using this framework for early-stage process optimization. Nonetheless, broader validation is essential for clinical relevance, particularly in medical applications where dimensional accuracy is critical. This limitation has been noted, and future work will incorporate expanded sampling to strengthen statistical power and application credibility.

Prior studies on bone screw fabrication have primarily focused on material biocompatibility, mechanical performance, or additive manufacturing techniques. Systematic investigations into the influence of injection molding parameters on the dimensional fidelity of cannulated bone screws remain scarce. In this context, the present study introduced a tailored AI-assisted optimization framework for hollow implant geometries, representing the first application of BPNN–GA–PSO modeling in this domain. The use of the normalized shrinkage rate as a predictive target reflects a focused adaptation of architecture to meet dimensional fidelity requirements in medical micromolding. Although each formulation was validated using three specimens, the observed consistency in shrinkage behavior supports the feasibility of using this approach for early-stage process screening. These findings lay the groundwork for future scale-up and broader clinical validation.

#### 3.3.2. Training and Prediction Performance

**Figure 15:**
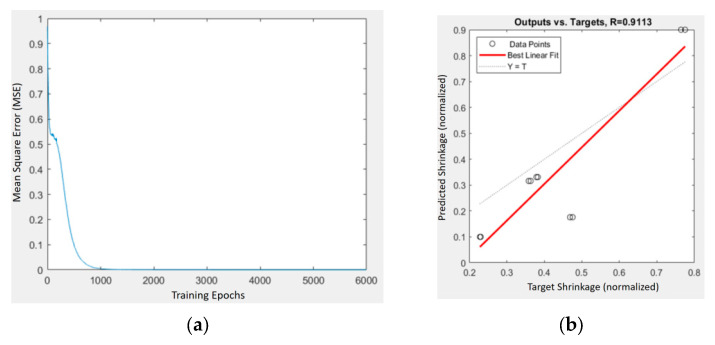
Training and prediction results of the BPNN model for PLA/PCL/HA composites: (**a**) MSE vs. iterations; (**b**) predicted vs. target shrinkage values with regression fit (R = 0.9113).

**Figure 16:**
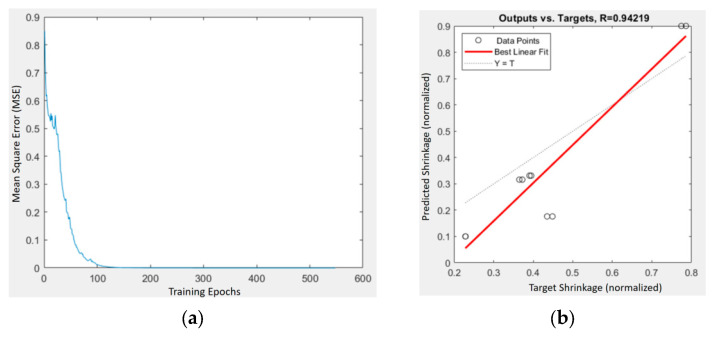
Training and prediction results of the BPNN model for PLA/PCL/TiO_2_ composites: (**a**) MSE vs. iterations; (**b**) predicted vs. target shrinkage values with regression fit (R = 0.94219).

Figure 15 and Figure 16 depict the training performance of the BPNN shrinkage predictors for the HA- and TiO_2_-enhanced composites, respectively. The PLA/PCL/HA model exhibited strong predictive accuracy, characterized by a low mean square error (MSE) and a high correlation coefficient (R), indicating effective mapping between input parameters and shrinkage outcomes.

Interestingly, the PLA/PCL/TiO_2_ network demonstrated even greater predictive stability and convergence. This enhanced response sensitivity may reflect a stronger coupling between process parameters and shrinkage behavior, suggesting that the molding conditions exert a more systematic influence on the TiO_2_ composite.

### 3.4. GA + PSO Optimization Results

#### 3.4.1. Optimization Setup

Building on the BPNN-based shrinkage predictors developed in Section 2.5, a hybrid genetic algorithm–particle swarm optimization (GA–PSO) strategy was implemented to identify optimal combinations of process parameters that minimize predicted shrinkage.

The objective function was defined to reduce deviation from a normalized shrinkage target of 0.1. Optimization was terminated when the best fitness value remained constant over successive generations, indicating convergence.

**Figure 17:**
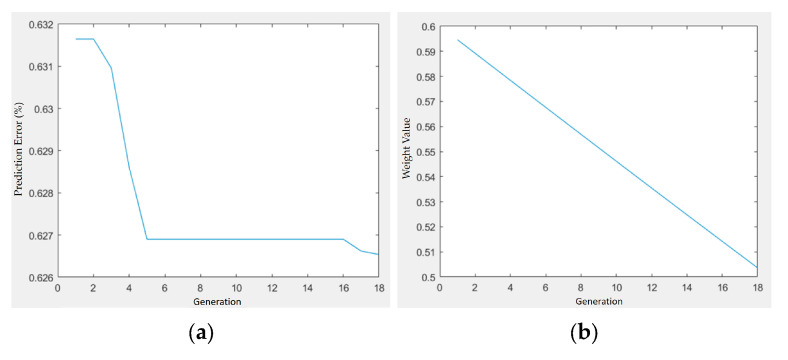
Optimization trajectory and convergence behavior of GA–PSO for shrinkage minimization in PLA/PCL/HA composites: (**a**) prediction error vs. generation; (**b**) weight value vs. generation.

**Figure 18:**
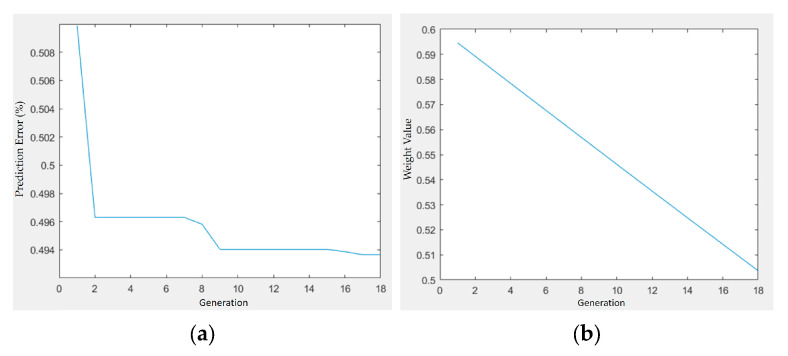
Optimization trajectory and convergence behavior of GA–PSO for shrinkage minimization in PLA/PCL/TiO_2_ composites: (**a**) prediction error vs. generation; (**b**) weight value vs. generation.

Figure 17 and Figure 18 present the optimization trajectories for the PLA/PCL/HA and PLA/PCL/TiO_2_ systems, respectively. The best fitness values showed different convergence speeds, with the HA system stabilizing earlier than the TiO_2_ system.

In both cases, the weight values assigned to the filler content (HA or TiO_2_) decreased gradually over generations, from approximately 0.595 to 0.505. This consistent trend may reflect the algorithm’s adjustment of parameter importance during optimization, though further analysis would be required to confirm its significance.

#### 3.4.2. Experimental Results and Validation

The optimal process parameter combinations determined using the GA–PSO strategy for both composite formulations are summarized in Table 14. These parameters were applied in injection molding trials to fabricate cannulated bone screws, and the resulting shrinkage rates were measured.

**Figure 19:**
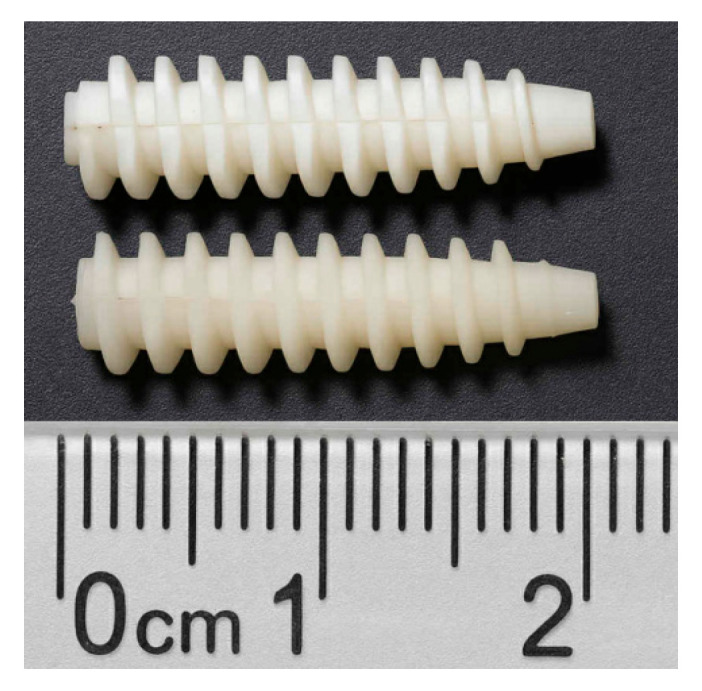
Molded PLA/PCL/TiO_2_ bone screw fabricated under GA–PSO-optimized conditions. The image shows the cannulate bone screw produced using the best parameter combination identified via BPNN-guided GA–PSO optimization. A quantitative comparison of shrinkage performance is provided in Table 15.

The dimensional results and average shrinkage for each formulation are listed in Table 15. The average shrinkage for the PLA/PCL/HA system was 2.838%, while that for the PLA/PCL/TiO_2_ system was lower—1.875%. The TiO_2_ formulation not only exhibited lower shrinkage but also demonstrated tighter dimensional control across all measured features, indicating enhanced process stability. This value is significantly lower than the minimum shrinkage observed in the original Taguchi experiments—3.44% for HA (L6) and 0.248% for TiO_2_ (L9). The molded screws with optimal parameters selected via AI are shown in Figure 19. The whiter one on top was made with the TiO_2_ formula, while the other one used the HA formula.

This outcome highlights the capability of the intelligent optimization framework to identify superior parameter combinations beyond the initial experimental space. These findings also support the applicability of the intelligent optimization workflow in guiding process design for biodegradable composite systems, offering a data-driven pathway to refine molding conditions beyond traditional design-of-experiment approaches.

#### 3.4.3. Influence of Temperature on Viscosity and Shrinkage Behavior

To address the observed difference in optimal processing conditions between the PLA/PCL/HA and PLA/PCL/TiO_2_ composites identified using the Taguchi method (A2 vs. A3), the following section discusses the rheological and interfacial characteristics of the respective fillers from a materials engineering perspective.

The ANOVA results derived from the Taguchi method revealed that melt temperature was the processing factor that most significantly influenced shrinkage rates for both the HA- and TiO_2_-filled PLA/PCL systems. This finding aligns well with the rheological behavior of the base PLA/PCL (80:20) matrix, which exhibits temperature-dominated viscosity variation under low shear conditions. As shown in Figure 12, across the shear rates relevant to injection molding of the cannulated bone screw, which were typically below 50 s^−1^ in thick-wall regions and approached 1000 s^−1^ in thin-wall segments, viscosity decreased with increasing temperature, whereas shear rate exerted only a limited effect.

Despite the similar temperature dependence observed in both filler systems, the optimal melt temperature differed: HA-filled composites performed best at 200 °C (A2), whereas TiO_2_-filled systems had a higher optimal temperature of 210 °C (A3). This difference can be attributed to the distinct particle characteristics of the fillers, as described below.

According to Tang et al. [8] and Luyt et al. [9], TiO_2_ nanoparticles (~100–300 nm) possess a high surface area and moderate polarity, which enhance crystallization and increase melt viscosity at elevated temperatures. Moreover, the high specific surface energy of untreated TiO_2_ tends to promote particle agglomeration, increasing flow resistance and requiring higher processing temperatures to achieve sufficient dispersion. These observations are consistent with the findings of Kaseem et al. [10], who reported poor distribution of untreated TiO_2_ in PLA matrices unless they were surface-modified.

In contrast, HA particles (~900 nm), as described by Hassanajili et al. [33] and RaziyanBukhari et al. [34], have larger particle sizes, lower surface areas, and lower interfacial energy with the polymer matrix. Although HA is also polar and prone to some agglomeration, its larger particle size and weaker matrix interaction result in less dramatic viscosity increases, making it more compatible with lower processing temperatures.

Interestingly, although the TiO_2_ formulation contained only 3 wt% filler compared to 10 wt% in the HA system, its impact on melt viscosity and flowability was more pronounced. This highlights the critical role of surface characteristics and interfacial interactions over filler concentration in determining the rheological behavior of polymer composites [8,9,10,33,34].

The strong temperature dependence of viscosity was also quantitatively captured in the modified cross model fitting of the PLA/PCL base resin, which showed a pronounced Arrhenius-type thermal sensitivity. The calculated activation energy (E_a_) was 80 kJ/mol, further confirming that thermal energy is a key driver in overcoming molecular and particulate resistance to flow in this system.

In summary, the results in Figure 12, combined with the mold-filling simulations (Figure 6), highlight that melt temperature exerts a stronger influence than shear rate on the flowability of the PLA/PCL-based biocomposites under the studied injection molding conditions. Consequently, thermal control should be prioritized over shear rate when selecting processing parameters to ensure the successful fabrication of micro-featured medical implants.

Taken together, these findings reinforce the commonly adopted strategy in thin-walled injection molding—using elevated melt temperatures to reduce viscosity—while also highlighting the need for formulation-specific adjustments based on filler characteristics. For cannulated bone screw geometry, where precise packing and thin-wall filling are critical, optimizing melt temperature remains essential due to the limited shear-induced viscosity modulation achievable within the actual mold shear rate profile.

#### 3.4.4. Composite Comparison and Material Recommendation

The shrinkage behavior of PLA/PCL/HA and PLA/PCL/TiO_2_ composites was systematically compared using GA–PSO-optimized process parameters, as summarized in Table 15. The TiO_2_-based system consistently exhibited lower shrinkage rates, with an average rate of 1.875%, compared to 2.838% for the HA-based composite. The TiO_2_-based composite showed a slightly lower average shrinkage rate (1.875%) than the HA-based system (2.838%).

This disparity is likely influenced by the combined effects of filler thermal conductivity and loading ratio during injection molding. TiO_2_ was incorporated at a lower concentration (3 wt%) but possesses a significantly higher thermal conductivity (~8.4 W/m·K), which may have facilitated more efficient heat dissipation and contributed to faster, more uniform cooling [8,10]. In contrast, the HA-based composite contained a higher filler content (10 wt%) and utilized a material with a much lower thermal conductivity (~1 W/m·K), limiting its ability to promote heat transfer during cooling. These differences in thermal transport characteristics may have contributed to the observed variation in shrinkage behavior, independent of filler volume fraction.

From a manufacturing perspective, the TiO_2_ composite demonstrated superior compatibility with precision molding applications, such as cannulated bone screws, where dimensional accuracy is critical. The HA composite, while less stable during shrinkage, remains advantageous for applications emphasizing bioactivity and osteoconductivity.

In summary, PLA/PCL/TiO_2_ is recommended for geometrically demanding applications, whereas PLA/PCL/HA may be more suitable for bioresorbable implants, where biological performance outweighs dimensional precision.

### 3.5. Recommendations Based on Comparative Analysis and Application

The comparative evaluation of PLA/PCL/HA and PLA/PCL/TiO_2_ systems revealed distinct differences in shrinkage behavior, thermal response, and predictive stability. While both systems exhibited acceptable mechanical integrity, the TiO_2_-enhanced composites demonstrated significantly lower shrinkage rates and reduced variability across mold temperature conditions. This dimensional stability is critical for applications requiring tight tolerances and reproducible geometries.

The HA system, despite its bioactivity, showed higher shrinkage fluctuations, which may limit its suitability for precision molding workflows. In contrast, the TiO_2_ system maintained consistent shrinkage performance and smoother process convergence, suggesting a more controllable behavior under molding conditions. These observations align with previous reports on TiO_2_’s stabilizing effect on polymer matrices [8], particularly in reducing rheological variability and shrinkage under dynamic processing conditions. The thermal responsiveness of TiO_2_ in PLA/PCL matrices has also been shown to enhance crystallization behavior and reduce thermal distortion [9].

An optimization framework based on hybrid AI strategies such as GA–PSO and BPNN was employed in this study to identify suitable process parameters for both formulations. While the model was trained on a unified dataset, its ability to accommodate formulation-specific responses highlights its utility as a process design tool. Rather than explaining the material differences, the AI framework facilitated efficient exploration of the parameter space, supporting reproducible molding outcomes and dimensional control [17].

These findings provide a foundation for the comparative application analysis presented in Section 3.4.2, where the distinct functional roles of HA and TiO_2_ fillers are evaluated in the context of bioresorbable implant design.

#### 3.5.1. Gating Strategy for Cannulated Bone Screw

To accommodate the unique geometric and clinical requirements of the cannulated bone screw, a gating strategy that diverges from conventional injection molding principles was adopted. The proximal segment, which is responsible for torque transmission during surgical insertion, bears the majority of axial and rotational loads. Gating through this region would compromise structural integrity and obstruct mold release.

While standard protocols favor gating through thicker sections to facilitate melt flow and shrinkage compensation [35], this study prioritized mechanical and surgical functionality. Consequently, the gate was repositioned to the thinner segment opposite the socket (wall thickness ≈ 0.4 mm), preserving the integrity of the load-bearing region.

As previously described in Section 2.4, a pin gate initiates melt flow and enables automatic runner separation in a three-plate mold. The melt is then directed through a diaphragm gate at the thin-wall region, promoting symmetric flow and cavity balance. Although manual removal of the diaphragm gate is required post-molding, this configuration maintains pressure continuity during packing and supports structural preservation.

Despite the challenges of gating in thin regions—such as early gate freeze-off—this approach protects critical geometry. Similar strategies have been documented for hollow or tubular components. Chen and Turng [36] emphasized that the gating location influences flow balance and functional geometry in thin-walled medical devices. Likewise, EP1504870A2 [37] describes symmetrical gating to ensure uniform filling and core alignment in catheter molding.

Thread profile geometry further contributes to molding feasibility. Bilateral crest flattening was applied along the threaded segment, extending to the root, facilitating demolding in micro-injection setups while preserving the flank geometry essential for torque transmission. Although Yu et al. [21] did not directly examine crest flattening, their findings underscore the importance of thread depth for torque capacity, implying that selective crest modifications are mechanically tolerable. Additionally, the flattened flanks may serve as passive retention pockets for autologous bone debris during insertion. Coyac et al. [38] confirmed that such retained fragments preserve osteoprogenitor viability and promote osteoinductive signaling, suggesting that intentional thread shaping may support biological integration.

Clinical guidelines further validate this configuration. According to Arthrex’s surgical protocol [39], proximal threads absorb primary axial and torsional loads, while the thinner segment opposite the socket primarily assists in positioning. This functional loading profile supports the gate placement and molding adaptations presented herein.

#### 3.5.2. Comparing the Suitability of PLA/PCL/HA and PLA/PCL/TiO_2_ Systems for Applications

The comparative evaluation of PLA/PCL composites reinforced with HA and TiO_2_ revealed a clear divergence in application suitability, driven by the distinct functional roles of each filler. TiO_2_-reinforced systems exhibited superior dimensional stability, with notably lower shrinkage rates and more consistent rheological behavior across processing conditions. These attributes suggest that TiO_2_ is advantageous in applications where geometric precision and formability are critical, such as in minimally invasive implant designs or components requiring tight tolerances.

In contrast, HA-reinforced composites exhibited slightly higher shrinkage and less consistent rheological behavior compared to their TiO_2_ counterparts. Nonetheless, their intrinsic bioactivity presents compelling advantages for osteoconductive applications, supporting their suitability for bioresorbable implant use. For anterior cruciate ligament (ACL) reconstruction, where bioresorbable cannulated bone screws must support bone ingrowth and gradual resorption, HA remains the clinically preferred additive. Long-term follow-up studies have confirmed that PLLA–HA screws promote favorable osteointegration and complete resorption, with minimal tunnel widening and stable fixation outcomes [40,41].

These findings suggest that while TiO_2_ may enhance processing reliability, its limited biological functionality may constrain its use in bone-interfacing applications. Conversely, HA offers superior biological performance, even though its rheological metrics are less favorable. Future composite designs may benefit from hybrid reinforcement strategies that integrate both fillers, aiming to balance mechanical stability with osteointegration potential—an approach that reflects the evolving demands of bioresorbable implant engineering.

Beyond the PLA/PCL-based systems studied here, the AI-assisted shrinkage optimization strategy—integrated with geometry-specific gate design and normalized modeling—offers a transferable framework for other bioresorbable polymers and hollow implant geometries requiring high-dimensional fidelity. This broader applicability underscores the potential of combining material-specific insights with process-aware modeling to support next-generation implant designs across diverse clinical contexts.

## 4. Conclusions

This study systematically investigated the mechanical and rheological properties of PLA/PCL composites reinforced with hydroxyapatite (HA) and titanium dioxide (TiO_2_), supplemented by thermal conductivity analysis, to aid interpretation of shrinkage behavior. The experimental results showed that TiO_2_-reinforced systems exhibited lower shrinkage rates and favorable molding outcomes, suggesting improved formability under the tested conditions. Although direct rheological measurements were not taken, the observed process consistency implies potential advantages in flow behavior.

In contrast, HA-reinforced composites demonstrated enhanced bioactivity, supporting their suitability for osteoconductive applications. Despite exhibiting slightly higher shrinkage and variable molding responses, these systems remain promising for clinical scenarios prioritizing biological integration.

AI-driven optimization using a BPNN and GA–PSO yielded reliable predictive models for shrinkage behavior, aligning well with experimental trends and providing a robust framework for future material design. The integration of empirical data with computational modeling underscores the potential of hybrid reinforcement strategies to balance mechanical performance with biological functionality.

Overall, the findings support the selective use of HA and TiO_2_ fillers based on specific clinical priorities and suggest that future composite designs may benefit from synergistic combinations to meet the evolving demands of bioresorbable implant engineering.

## Figures and Tables

**Table 1:** L9 Orthogonal array for injection molding parameters used in bPNN training.

Level	Melt Temperature (°C)	Injection Speed (mm/s)	Holding Speed (mm/s)
1	190	40	10
2	200	60	20
3	210	80	30

**Table 2:** Fixed injection molding parameters used in BPNN training.

Parameters	Numerical Value
Mold Temp	30 °C
Cooling Time	15 s
Holding Time	3 s
Back Pressure	40 bar
Screw Speed	80 rpm
Metering Volume	470 mm^3^

**Table 3:** Experimental runs based on L9 orthogonal array for BPNN training. Nine combinations of melt temperature, injection speed, and holding speed were arranged using an L9 orthogonal array to generate uniformly distributed training data and assess the contributions of various factors to product quality.

Run	Melt Temperature (°C)	Injection Speed (mm/s)	Holding Speed (mm/s)
1	190	40	10
2	190	60	20
3	190	80	30
4	200	40	20
5	200	60	30
6	200	80	10
7	210	40	30
8	210	60	10
9	210	80	20

**Table 6:** ANOVA results for shrinkage variation (PLA/PCL/HA).

Control Factor	Variance (S)	DoF (f)	Mutations (V)	Pure Change (S)	Contribution (ρ)%
A (Melt Temperature)	25.389	2	12.695	23.764	37.70
B (Injection Speed)	16.458	2	8.229	14.833	24.44
C (Holding Speed)	9.247	2	4.624	7.622	13.73
e (Error)	16.247	20	0.812	0	24.13
Total	67.337	26		45.674	100

**Table 7:** Dimensional measurements of PLA/PCL/TiO_2_ bone screws (unit: mm).

Trial	Specimen	Length	Thread Outer Diameter	Bore Diameter	Hex Socket Width
L1	#1	19.647	5.92	1.673	2.449
	#2	19.685	5.935	1.693	2.426
	#3	19.662	5.896	1.671	2.428
L2	#1	19.479	5.883	1.68	2.363
	#2	19.637	5.893	1.668	2.335
	#3	19.472	5.957	1.674	2.321
L3	#1	19.71	5.885	1.66	2.431
	#2	19.796	5.955	1.667	2.431
	#3	19.778	5.874	1.667	2.345
L4	#1	19.763	5.891	1.675	2.282
	#2	19.822	5.936	1.67	2.393
	#3	19.906	5.882	1.669	2.378
L5	#1	19.725	5.863	1.652	2.331
	#2	19.682	5.897	1.678	2.324
	#3	19.628	5.858	1.668	2.386
L6	#1	19.969	5.934	1.653	2.443
	#2	19.907	5.948	1.673	2.385
	#3	19.848	5.878	1.66	2.418
L7	#1	19.945	5.853	1.659	2.422
	#2	19.964	5.928	1.66	2.413
	#3	19.943	5.865	1.672	2.477
L8	#1	19.943	5.936	1.67	2.449
	#2	19.823	5.908	1.683	2.43
	#3	19.782	5.945	1.663	2.393
L9	#1	19.727	5.856	1.686	2.383
	#2	19.827	5.894	1.69	2.403
	#3	19.822	5.884	1.683	2.394

**Table 8:** Summary of shrinkage rates, standard deviations, shrinkage–variation sums, and S/N ratios for PLA/PCL/TiO_2_ composite trials.

Run	Total Shrinkage (%)	Total SD(%)	Shrinkage + SD (%)	S/N
L1	7.029	1.64	8.669	23.062
L2	11.859	2.337	14.196	18.519
L3	8.873	3.189	12.062	21.038
L4	10.217	3.439	13.656	19.815
L5	12.009	2.731	14.74	18.409
L6	7.509	2.679	10.188	22.488
L7	6.914	2.555	9.469	23.205
L8	6.086	2.478	8.564	24.313
L9	4.248	1.215	5.463	27.436

**Table 9:** ANOVA results for shrinkage variation (PLA/PCL/TiO_2_).

Control Factor	Variance (S)	DoF (f)	Mutations (V)	Pure Change (S)	Contribution (ρ)%
A (Melt Temperature)	39.853	2	19.926	39.443	58.25
B (Injection Speed)	15.749	2	7.875	15.339	23.02
C (Holding Speed)	8.717	2	4.359	8.307	12.74
e (Error)	4.104	20	0.2052	0	6.00
Total	68.426	26		63.089	100

**Table 10:** Setting the parameters of the back-propagation neural network.

Input Layer	Three Neurons
Hidden Layer	One layer, eight neurons
Learning Rate	0.01
Normalization range	0.1~0.9
Activation function	Sigmoid function
Convergence Threshold	10^−7^
Training maximum epochs	12,000

**Table 11:** Verification group parameters. Each group (#1–#5) included three molding parameters—melt temperature, injection speed, and holding speed—for both PLA/PCL/HA and PLA/PCL/TO_2_ systems.

	PLA/PCL/HA			PLA/PCL/TO_2_		
Run	Melt Temp. (°C)	Injection Speed (mm/s)	Holding Speed (mm/s)	Melt Temp. (°C)	Injection Speed (mm/s)	Holding Speed (mm/s)
#1	194	80	30	190	63	30
#2	210	53	14	194	40	10
#3	202	60	10	210	74	23
#4	206	40	26	202	80	30
#5	190	46	18	198	51	16

**Table 12:** PLA/PCL/HA back-transfer neural network normalization data.

		Melt Temp	Injection Speed	Holding Speed	Shrinkage
Training Group	L1	0.100	0.100	0.100	0.730
	L2	0.100	0.500	0.500	0.900
	L3	0.100	0.900	0.900	0.785
	L4	0.500	0.100	0.500	0.871
	L5	0.500	0.500	0.900	0.352
	L6	0.500	0.900	0.100	0.100
	L7	0.900	0.100	0.900	0.656
	L8	0.900	0.500	0.100	0.818
	L9	0.900	0.900	0.500	0.521
Test Group	Test 1	0.260	0.900	0.900	0.854
	Test 2	0.900	0.367	0.260	0.534
	Test 3	0.580	0.500	0.100	0.900
	Test 4	0.740	0.100	0.740	0.900
	Test 5	0.100	0.233	0.420	0.100

**Table 13:** PLA/PCL/TiO_2_ back-transfer neural network normalization data.

		Melt Temp	Injection Speed	Holding Speed	Shrinkage
Training Group	L1	0.100	0.100	0.100	0.387
	L2	0.100	0.500	0.500	0.885
	L3	0.100	0.900	0.900	0.577
	L4	0.500	0.100	0.500	0.715
	L5	0.500	0.500	0.900	0.900
	L6	0.500	0.900	0.100	0.436
	L7	0.900	0.100	0.900	0.375
	L8	0.900	0.500	0.100	0.289
	L9	0.900	0.900	0.500	0.100
Test Group	Test 1	0.100	0.557	0.900	0.100
	Test 2	0.260	0.100	0.100	0.706
	Test 3	0.900	0.786	0.633	0.900
	Test 4	0.580	0.900	0.900	0.827
	Test 5	0.420	0.329	0.367	0.730

**Table 14:** GA–PSO-optimized process parameters for PLA/PCL/HA and PLA/PCL/TiO_2_ formulas used in injection molding trials.

		Melt Temperature	Injection Speed	Holding Speed
HA	Normalized Value	0.4024	0.7472	0.2932
	Actual Value	197.56 °C	72.36 mm/s	14.83 mm/s
TiO_2_	Normalized Value	0.7548	0.8362	0.6448
	Actual Value	206.37 °C	76.81 mm/s	23.62 mm/s

**Table 15:** Dimensional measurements and average shrinkage rates of bone screws fabricated under AI-optimized molding conditions for HA and TiO_2_ systems. Measurements were obtained for three samples per material system. The dimensional consistency and shrinkage rates reflect the effectiveness of AI-driven process optimization.

HA	Length (mm)	Thread Outer Diameter (mm)	Bore Diameter (mm)	Hex Socket Width (mm)	Average Shrinkage (%)
#1	19.941	5.929	1.693	2.494	2.238
#2	19.985	5.966	1.677	2.468	3.383
#3	19.888	5.964	1.688	2.477	2.893
Average	19.938	5.953	1.686	2.480	2.838
**TiO_2_**	**Length (mm)**	**Thread Outer Diameter (mm)**	**Bore Diameter (mm)**	**Hex Socket Width (mm)**	**Average Shrinkage (%)**
#1	19.988	5.928	1.699	2.486	1.986
#2	19.984	5.962	1.675	2.497	2.412
#3	19.966	5.982	1.691	2.497	1.226
Average	19.979	5.957	1.688	2.493	1.875

## Data Availability

The original contributions presented in the study are included in the article, further inquiries can be directed to the corresponding author.
